# Enlarged perivascular spaces are associated with the disease activity in systemic lupus erythematosus

**DOI:** 10.1038/s41598-017-12966-4

**Published:** 2017-10-03

**Authors:** Mari Miyata, Shingo Kakeda, Shigeru Iwata, Shingo Nakayamada, Satoru Ide, Keita Watanabe, Junji Moriya, Yoshiya Tanaka, Yukunori Korogi

**Affiliations:** 1 Department of Radiology, School of Medicine, University of Occupational and Environmental Health, Kitakyushu, Fukuoka Japan; 2 The First Department of Internal Medicine, School of Medicine, University of Occupational and Environmental Health, Kitakyushu, Fukuoka Japan

## Abstract

To determine whether any brain MR abnormalities, including enlarged perivascular spaces (EPVS), were associated with disease activity in systemic lupus erythematosus (SLE) as an inflammatory activity. One hundred and thirty SLE patients with normal MR findings were assessed. With regard to MRI abnormalities, patients with brain atrophy and mild white matter hyperintensity (WMH) on T2WI were not excluded. The disease activity was assessed using the SLEDAI and the BILAG scores. The imaging characteristics included centrum semiovale EPVS (CS- EPVS) and basal ganglia EPVS on T2WI, WMH, and brain atrophy. We used univariate and multivariate logistic regression analyses to determine the clinical (vascular risk factors and blood examinations) and imaging characteristics that were associated with the disease activity of SLE. High CS-EPVS to be the only factor that was independently associated with the severity of the SLEDAI and BILAG scores (odds ratio [OR] 5.77; 95% confidence interval [CI] 2.21–15.00; p < 0.001 for the SLEDAI, and OR 2.64; 95% CI 1.03–6.74; p = 0.042 for the BILAG score). The CS-EPVS in the SLE patients are associated with the systemic disease activity, suggesting that CS- EPVS may be indicative of the reactive changes of the white matter due to the inflammatory activity.

## Introduction

Systemic lupus erythematosus (SLE) is an autoimmune disease that frequently manifests with central nervous system (CNS) involvement^1,2^. The main primary pathophysiology of the brain in SLE patients is reported to be inflammation that occurs secondarily to the autoantibody-mediated effects in the brain tissue^3^; however, the etiology remains uncertain. Many MRI studies have supplied ample evidence of brain pathology in SLE patients, such as cerebral infarction, brain atrophy and multifocal gray matter (GM) and/or white matter (WM) lesions^4^. However, these MR findings may indicate that the pathological conditions of the brain occur as a result of SLE-related inflammation. To our knowledge no MRI studies have been performed to assess whether inflammatory activity incites the primary pathology of SLE.

The perivascular spaces (PVSs) are cerebrospinal fluid-filled cavities that surround small penetrating cerebral arterioles in the centrum semiovale (CS), basal ganglia (BG), and hippocampus. Enlarged PVSs (EPVS) are a common finding on brain MRI but their pathophysiological significance is unclear and the finding is generally overlooked. EPVS are not usually present in the brains of healthy young adults, but become increasingly common with ageing^5^. Recently, EPVS have been a focus of research as an MRI marker of neuroinflammatory activity in the brain. In previous MRI studies^6–8^, EPVS are observed in various inflammatory disorders, including multiple sclerosis (a chronic inflammatory disease of the CNS), and experimental autoimmune encephalomyelitis. These findings suggest that PVS are sites in which inflammatory activity is triggered in the CNS, and that EPVS may thus play a role in the onset of neuroinflammatory activity. Accordingly, we hypothesized that the inflammatory activity, which leads to the infiltration of cells in small vessels, may also lead to the development of EPVS in SLE patients; thereby resulting in an increased number of EPVS (which are detectable by MRI). To the best of our knowledge, no previous studies have investigated the presence of EPVS in SLE patients.

In previous studies with SLE patients, the inflammatory activity was demonstrated to be associated with clinical disease activity of SLE; the markers of inflammation (the erythrocyte sedimentation rate: ESR)^9,10^ and proinflammatory cytokines (IL-6)^11^ were associated with Systemic Lupus Erythematosus Disease Activity Index (SLEDAI) score. Thus, the purpose of this study was to determine whether any MR abnormalities, including EPVS, were associated with the disease activity in SLE as an inflammatory activity.

## Methods

### Approval

Human experiments were carried out in accordance with the guidelines provided and approved by the Institutional Review Board of the University of Occupational and Environmental Health School of Medicine (Kitakyushu, Fukuoka, Japan). The need to obtain informed consent from the patients was waived.

### Subjects

**Figure 1:**
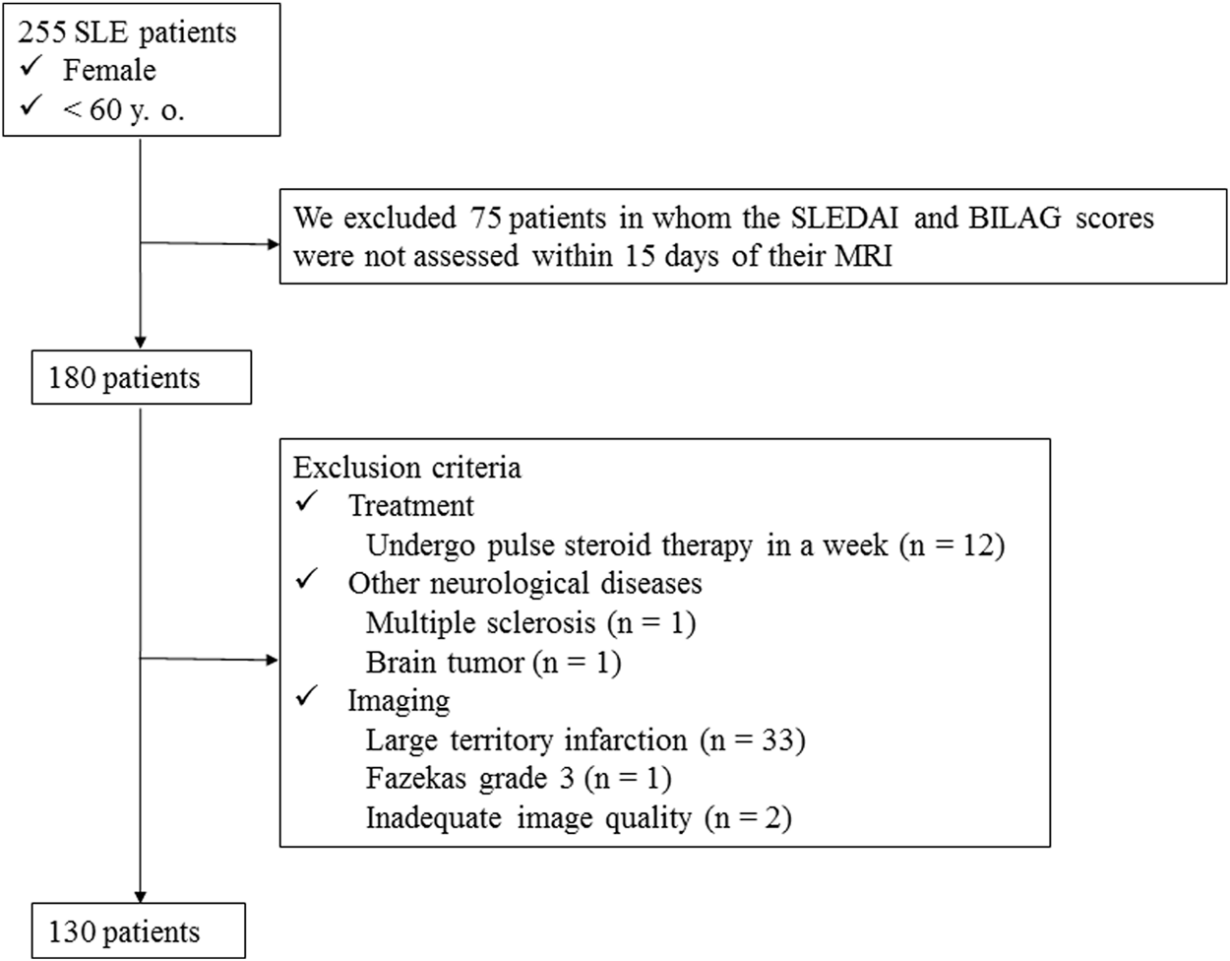
Flow chart of patient.

We routinely perform screening brain MRI studies to assess patients with SLE. In the present analysis, we included patients who were younger than 60 years of age to avoid the potential age-related bias posed by cerebrovascular disease^12^. Thus, one radiologist (M.M. with 6 years of neuroradiology experience) reviewed the MRI database of patients who were examined between May 2004 and Feb 2017 and selected 255 female patients who were younger than 60 years of age, who had been diagnosed with SLE according to the American Rheumatism Association criteria for the classification of SLE^13^ (Fig. 1). The disease activity of SLE patients is quantified using a Safety of Estrogens in Lupus Erythematosus National Assessment–SLE Disease Activity Index (SELENA-SLEDAI) score^14^ and the British Isles Lupus Assessment Group 2004 (BILAG) index score^15^. From these patients, we further selected 180 patients in whom disease activity was assessed within 15 days of their MRI examinations using the SLEDAI and BILAG scores.

The exclusion criteria included patients who were currently undergoing pulse steroid therapy or those who were in the 1st week after treatment, patients with a history of other neurological diseases, unsatisfactory images due to artifacts, and/or MRI abnormalities. With regard to abnormal MR findings, patients with brain atrophy and mild WM hyperintensity (WMH) (Fazekas grade 1) on T2-weighted imaging (T2WI)/ fluid-attenuated inversion recovery (FLAIR) imaging^16^ were not excluded. Thus, we excluded 12 patients who were undergoing pulse steroid therapy or who were in the 1st week after treatment, 2 patients because they had a brain tumor or multiple sclerosis, and 2 patients in whom the image quality was inadequate. A total of 34 SLE patients were excluded based on the following MRI exclusion criteria: large territory infarction (n = 33), WMH (Fazekas grade 3; n = 1, grade 4; n = 0). Thus, a total of 130 female patients were included in the evaluation (Table 1).

**Table 1:** Imaging and clinical characteristics of SLE and healthy subjects.

	SLE	control	p value
	n = 130	n = 40	
**Characteristics**			
Age (ave. ± SD)	36.1 ± 12.0	35.0 ± 10.9	0.781
Smoking, %	15 (12)	2 (5)	0.259
Obesity, %	11 (8)	0 (0)	—
Duration (month ± SD)	46.9 ± 115.5	—	—
**Imaging**			
Atrophy, %	32 (25)	0 (0)	<0.001
WML, %	46 (35)	1 (3)	<0.001
High CS-EPVS (>20), %	75 (58)	4 (10)	<0.001
High BG-EPVS (>20), %	23 (18)	1 (3)	<0.001
**Medical history**			
Pulse steroid therapy, %	28 (22)	—	—
Average PSL dose (mg ± SD)	5.8 ± 9.1	—	—
**Medical comorbidities**			
Hypertension, %	32 (25)	3 (8)	0.039
Hyperlipidemia, %	12 (9)	1 (3)	0.158
Diabetes mellitus, %	9 (7)	1 (3)	0.390
**Acivity index (ave. ± SD)**			
SLEDAI score	11.8 ± 7.6	—	—
BILAG index score	13.6 ± 9.0	—	—
nBILAG index score	1.29 ± 2.8	—	—
**Blood examinations (ave. ± SD)**			
WBC (10^3^/μL)	5.2 ± 9.1	—	—
PLT (10^3^/μL)	209.8 ± 87.6	—	—
ESR (mm/hr)	47.4 ± 33.4	—	—
CH50 (U/ml)	33.1 ± 17.1	—	—
Anti-dsDNA antibody (IU/ml)	54.9 ± 82.0	—	—

Abbreviations: WML = white matter lesion; CS = centrum semiovale; EPVS = enlarged perivascular spaces; BG = basal ganglia; PSL = prednisolone; SLEDAI = Systemic Lupus Erythematosus Disease Activity Index; BILAG = British Isles Lupus Assessment Group; nBILAG = neuro British Isles Lupus Assessment Group; WBC = white blood cell; PLT = platelet; ESR = erythrocyte sedimentation rate; CH50 = 50% hemolytic unit of complement; anti-dsDNA antibodie = anti-double stranded DNA antibody.

We selected 40 age- and sex-matched controls who from the same sample population, who had no history of neurological or psychiatric diseases and who underwent MRI between Jan 2008 and December 2008. The indications for their examination included screening, headaches or benign positional vertigo. The conventional MRI results were normal in all controls. As with the study subjects, patients with brain atrophy and WMH (Fazekas grade 1) on T2WI/FLAIR imaging^16^ were not excluded.

We reviewed the duration of SLE (interval between the initial onset of SLE and the brain MR study and demographic data for vascular risk factors (diabetes mellitus [defined as a random glucose level of >11.1 mmol/l, fasting blood glucose >7.0 mmol/l, HbA1c >6.5%, or the current use of antidiabetic drugs), hypertension (blood pressure >140/90 mm Hg or current treatment with antihypertensive drugs), past or current smoking, dyslipidemia (LDL cholesterol >3.64 mmol/l, HDL cholesterol <0.91 mmol/l, triglyceride > 1.7 mmol/l, or treatment for dyslipidemia), obesity (body mass index >26 kg/m^2^), and treatments (their history of pulse steroid therapy and their average prednisolone [PSL] dose). We also reviewed the white blood cell (WBC), platelet (PLT), ESR, 50% hemolytic unit of complement (CH50), and anti-double stranded DNA antibody (anti-dsDNA antibody) as a marker of the disease activities.

### Image acquisition

All studies were performed using the 3-T MRI system (Signa EXCITE 3 T; GE Healthcare) with a dedicated eight-channel phased-array coil (USA Instruments Aurora, OH, USA).

All patients and controls underwent brain MRI according to our standard protocol, including T2WI, FLAIR imaging and T1-weighted imaging (T1WI). T2WI, FLAIR imaging and T1WI (spin-echo imaging) were obtained on the axial planes. For T1WI (3D fast spoiled gradient-echo imaging), we used three cross-section images (coronal, axial, and sagittal) reconstructed from the images obtained on the sagittal plane. The imaging parameters (repetition time ms/echo time ms/inversion time/NEX/imaging time) were 4,500/85/NA/1/2 min and 10 s for T2WI, 12,000/140/2,600/2/3 min and 20 s for FLAIR imaging and 400/4/NA/2/2 min and 40 s for spin-echo T1WI. The T2WI, T1WI, and FLAIR images were acquired at a section thickness of 4 mm, an intersection gap of 2.5 mm, a field of view of 22 cm and a matrix of 256 × 192. The following parameters were used for spoiled gradient-echo imaging: repetition time ms/echo time ms, 10/4; flip angle, 10°; bandwidth, 42 kHz; section thickness, 1.2 mm; matrix, 256 × 256; field of view, 24 × 24 cm; imaging time, 3 minutes and 56 seconds.

### Image interpretation

**Figure 2:**
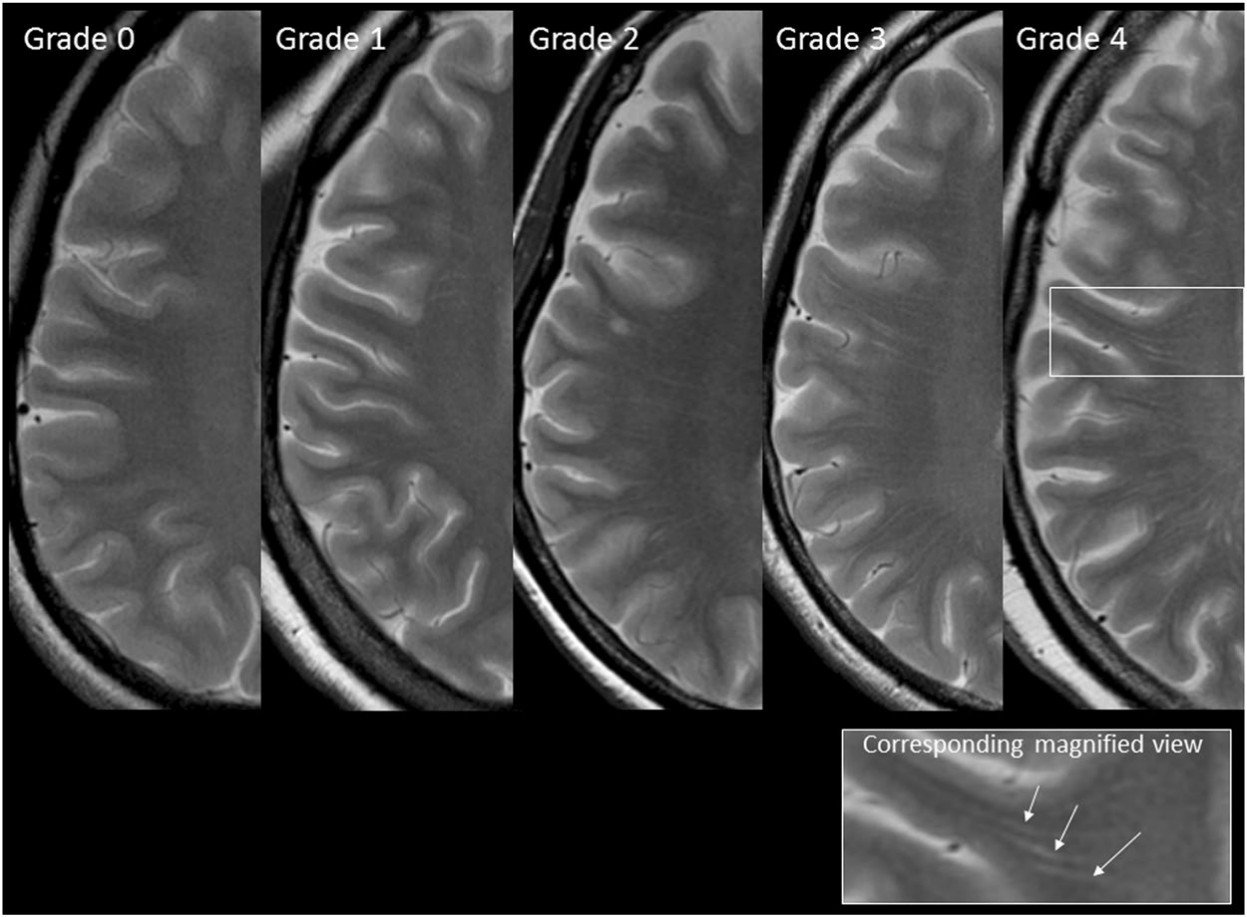
The grading scale for centrum semiovale EPVS (CS-EPVS); Grade 0, no EPVS; Grade 1, 1 to 10 EPVS; Grade 2, 11 to 20 EPVS; Grade 3, 21 to 40 EPVS; Grade 4, >40 EPVS. Corresponding magnified view depicting EPVS (arrows).

Two neuroradiologists (S.K and S.I), who were blind to the diagnosis and the clinical data, reviewed the MR studies and categorized the findings (brain atrophy, WMH, and EPVS) by consensus. Brain atrophy was defined as deep (enlargement of the ventricles) or peripheral (enlargement of the gyri) and rated on a subjective scale of 0 to 3 (0 = absent, 1 = mild, 2 = moderate, 3 = severe)^17^. WMH was graded from 0 to 3 according to the Fazekas scale (0 = none; 1 = single punctate; 2 = multiple punctate; early confluent; 3 = large confluent)^16^. EPVS were defined as small, sharply delineated structures of cerebrospinal fluid intensity on imaging that followed the orientation of the perforating vessels and ran perpendicular to the brain surface. Thus, they appeared round in axial sections (in the BG); they were linear if they appeared in longitudinal sections (in the CS) and were <3 mm in width^5,18^. They showed a high signal intensity on T2WI and a low signal intensity on T1WI and FLAIR, and had no mass effect. Only the areas that met all of the above-mentioned criteria were considered to be EPVS. The neuroradiologists assessed EPVS in the BG (Type I EPVS: identified along the lenticulostriate arteries going through the BG from the anterior perforated substance) and CS (type II EPVS: identified along the perforating medullary arteries as they penetrated the cortical GM over the high convexities and extended into the WM) separately because it is possible that their pathophysiology may have been distinct (due to their different locations and features). EPVS in both the BG and CS were coded with the following scale (which was applied to standard axial images): 0 = no EPVS, 1 = 1 to 10 EPVS, 2 = 11 to 20 EPVS, 3 = 21 to 40 EPVS, and 4 =  > 40 EPVS^5,19^ (Fig. 2). The numbers refer to EPVS on one side of the brain; the higher score was used if there was asymmetry between the sides and EPVS were counted in the slice with the highest number.

### Statistical analysis

Based on the SLEDAI and BILAG scores, we categorized the SLE patients into a severe group (SLEDAI score ≥11 or BILAG score ≥6) and a non-severe group (SLEDAI score, <11 or BILAG score, <6) according to the previous reports^20,21^. We also divided the patients into 2 groups according to their neuro BILAG (nBILAG) scores (A, B, and C vs D and E). With regard to the treatments, the patients were divided into 2 groups based on whether or not they had a history of pulse steroid therapy.

We defined high BG-EPVS and high CS-EPVS (grades 3–4) as the presence of >20 EPVS, in line with the most severe category of EPVS used in previous studies^22,23^. For the subjective scale of brain atrophy, “grades 3 and 4” were defined as pathologic brain atrophy. We also divided the patients into 2 groups based on the presence or absence of WMH (Fazekas grade 0 vs grade 1). For the analyses, the following parameters: age, duration of SLE, average PSL dose, WBC, PLT, ESR, CH50, and anti-dsDNA antibody were treated as a continuous variable.

We compared the clinical and imaging characteristics, including the EPVS between SLE patients and controls, and between the severe and non-severe groups (as assessed by the patients’ SLEDAI and BILAG scores) using the x^2^ test and Fisher’s exact test for categorical variables, and the 2-sample *t*-test or Mann-Whitney U test for continuous variables, as appropriate.

We used a univariate binary logistic regression analysis to determine the variables that were associated with the disease activity (SLEDAI and BILAG scores) and the number of EPVS. Variables with a *p* value of <0.1 on the univariate analysis were entered into multivariate logistic model. Multivariate analyses were performed by separating SLEDAI score from BILAG score because these scores are obvious confounding factors. *P* values < 0.05 were considered indicate statistical significance. All of the statistical analyses were performed using an application program “R” [R Development Core Team (2012). R: A language and environment for statistical computing. R Foundation for Statistical Computing, Vienna, Austria. ISBN 3-900051-07-0, http://www.R-project.org/].

## Result

Table 1 summarizes the clinical and radiological characteristics of the SLE patients and controls. Pathological brain atrophy (25% vs 0%; p < 0.001), high WMH scores (35% vs 3%; p < 0.001), high CS-EPVS (58% vs 10%; p < 0.001), and high BG-EPVS (23% vs 3%; p < 0.001) were more commonly seen in patients than in controls. The categories of CS-EPVS were as follows: category 0 (n = 5 vs 19), 1 (n = 50 vs 17), 2 (n = 42 vs 4), 3 (n = 29 vs 0), and 4 (n = 4 vs 0) in patients (n = 130) vs controls (n = 40). The categories of BG-EPVS were as follows: category 0 (n = 19 vs 15), 1 (n = 88 vs 24), 2 (n = 23 vs 1), 3 (n = 0 vs 0), and 4 (n = 0 vs 0) in the patients vs the controls.

**Figure 3:**
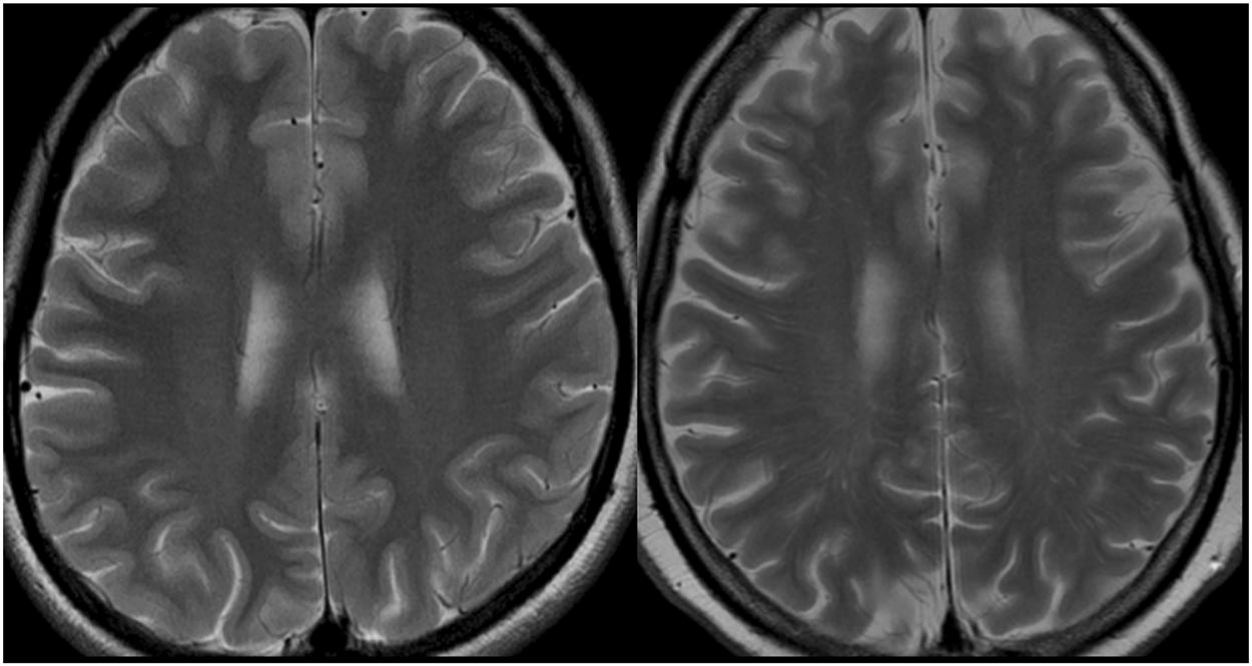
(**a**) A 30-year-old woman with mild SLE activity (SLEDAI score = 0, BILAG score = 1). (**b**) A 36-year-old woman with severe SLE activity (SLEDAI score = 12, BILAG score = 15). EPVS are not observed on T2WI (**a**) (CS-EPVS grade 0). A T2WI (**b**) shows that linear hyperintensity is a characteristic of EPVS in the centrum semiovale (CS-EPVS grade 4).

Table 2 summarizes the clinical and imaging characteristics for the subgroup comparisons in SLE patients. For the SLEDAI and BILAG scores, high CS-EPVS was significantly more prevalent in the severe group than in the non-severe group (SLEDAI score: 76% vs 34%; p < 0.001 and BILAG score: 66% vs 32%; p < 0.001) (Fig. 3), whereas there were no significant differences in the nBILAG score. Patients with a history of pulse steroid therapy were significantly more prevalent in the severe group than in the non-severe group (based on the SLEDAI score), and the average PSL dose was significantly higher in the severe group than in the non-severe group. With regard to the other imaging characteristics (pathologic brain atrophy, a high WMH score, and high BG-EPVS), there were no statistically significant differences between the severe and non-severe groups.

**Table 2:** The clinical and imaging characteristics for the subgroup comparisons in SLE patients.

	SLEDAI			BILAG			nBILAG		
	severe	non-severe	p value	severe	non-severe	p value	severe	non-severe	p value
	n = 72	n = 58		n = 99	n = 31		n = 33	n = 97	
**Characteristics**									
Age (ave. ± SD)	36.8 ± 11.9	35.3 ± 12.2	0.476	35.6 ± 11.6	37.7 ± 13.3	0.416	32.4 ± 9.1	37.5 ± 12.7	0.035
Smoking, %	7 (10)	8 (14)	0.474	13 (13)	2 (6)	0.313	4 (12)	11(11)	0.904
Obesity, %	4 (6)	7 (12)	0.187	6 (6)	5 (16)	0.080	2 (6)	9 (9)	0.570
Duration (month ± SD)	76.0 ± 92.1	52.0 ± 83.0	0.120	59.8 ± 89.4	72.3 ± 82.4	0.490	67.5 ± 78.9	61.1 ± 90.7	0.718
**Imaging**									
Atrophy, %	24 (33)	17 (29)	0.752	34 (34)	7 (23)	0.175	14 (42)	27 (28)	0.079
WML, %	28 (39)	18 (31)	0.356	37 (37)	9 (29)	0.401	11 (33)	35 (36)	0.777
High CS-EPVS (>20), %	55 (76)	20 (34)	<0.001	65 (66)	10 (32)	<0.001	21 (64)	54 (56)	0.428
High BG-EPVS (>20), %	14 (19)	9 (16)	0.563	19 (19)	4 (13)	0.427	5 (15)	18 (19)	0.661
**Medical history**									
Pulse steroid therapy, %	13 (18)	15 (26)	0.285	21 (21)	7 (23)	0.873	7 (21)	21 (22)	0.958
Average PSL dose (mg ± SD)	6.2 ± 13.6	5.4 ± 6.1	0.780	5.7 ± 9.4	6.1 ± 8.1	0.809	5.2 ± 7.5	5.9 ± 9.6	0.835
**Medical comorbidities**									
Hypertension, %	23 (32)	9 (16)	0.031	28 (28)	4 (13)	0.084	4 (12)	28 (29)	0.054
Hyperlipidemia, %	8 (11)	4 (7)	0.413	9 (9)	3 (10)	0.922	3 (9)	9 (9)	0.975
Diabetes mellitus, %	6 (8)	3 (5)	0.484	8 (8)	1 (3)	0.357	2 (6)	7 (7)	0.823
**Acivity index (ave. ± SD)**									
SLEDAI score	17.9 ± 5.6	4.7 ± 3.2	<0.001	14.3 ± 7.4	4.7 ± 5.1	<0.001	14.1 ± 9.0	11.3 ± 7.6	0.087
BILAG index score	18.0 ± 8.5	6.7 ± 5.3	<0.001	16.2 ± 8.1	2.6 ± 2.0	<0.001	18.1 ± 9.2	11.3 ± 8.2	<0.001
nBILAG index score	2.3 ± 3.8	0.7 ± 2.1	0.003	2.1 ± 3.6	0.2 ± 0.6	0.004	6.3 ± 3.4	0	—
**Blood examinations (ave. ± SD)**									
WBC (10^3^/μL)	5.0 ± 2.8	5.4 ± 2.4	0.330	5.1 ± 2.6	5.5 ± 2.7	0.497	5.0 ± 2.2	5.3 ± 2.8	0.596
PLT (10^3^/μL)	201.7 ± 95.3	219.8 ± 76.6	0.242	204.1 ± 91.7	227.8 ± 71.2	0.190	207.0 ± 97.2	210.7 ± 84.6	0.833
ESR (mm/hr)	56.8 ± 33.3	35.7 ± 29.8	<0.001	52.3 ± 33.9	31.6 ± 26.2	0.002	40.7 ± 29.8	49.6 ± 34.4	0.186
CH50 (U/ml)	26.7 ± 16.6	40.9 ± 14.4	<0.001	32.1 ± 17.4	36.0 ± 16.1	0.271	54.2 ± 83.7	32.5 ± 17.9	0.490
Anti-dsDNA antibody (IU/ml)	152.5 ± 161.3	31.2 ± 58.7	<0.001	117.6 ± 151.2	37.0 ± 64.1	0.004	94.5 ± 138.8	99.7 ± 140.6	0.855

Abbreviations: WML = white matter lesion; CS = centrum semiovale; EPVS = enlarged perivascular spaces; BG = basal ganglia; PSL = prednisolone; SLEDAI = Systemic Lupus Erythematosus Disease Activity Index; BILAG = British Isles Lupus Assessment Group; nBILAG = neuro British Isles Lupus Assessment Group; WBC = white blood cell; PLT = platelet; ESR = erythrocyte sedimentation rate; CH50 = 50% hemolytic unit of complement; anti-dsDNA antibodie = anti-double stranded DNA antibody.

Table 3 lists the results of the univariate and multivariate logistic analyses for disease activity. The severity, as indicated by the SLEDAI and BILAG scores, was independently associated with high CS-EPVS (p < 0.001 for SLEDAI and BILAG scores). The severity, as indicated by the SLEDAI score, was also independently associated with a presence of hypertension, ESR and CH50.

**Table 3:** The univariate and multivariate logistic analyses for activity index (SLEDAI, BILAG index, and nBILAG).

	SLEDAI				BILAG				nBILAG	
	univariate analysis		multivariate analysis		univariate analysis		multivariate analysis		univariate analysis	
	OR (95% CI)	p value	OR (95% CI)	p value	OR (95% CI)	p value	OR (95% CI)	p value	OR (95% CI)	p value
**Characteristics**										
Age	1.01 (0.98–1.04)	0.473	—	—	0.99 (0.95–1.02)	0.414	—	—	0.96 (0.93–1.00)	0.039
Smoking	0.67 (0.23–1.98)	0.472	—	—	2.19 (0.47–10.30)	0.320	—	—	1.08 (0.32–3.65)	0.903
Obesity	0.43 (0.12–1.54)	0.195	—	—	0.34 (0.09–1.19)	0.090	0.33 (0.08–1.47)	0.146	0.63 (0.13–3.08)	0.569
Duration	1.00 (0.99–1.00)	0.124	—	—	1.00 (0.99–1.00)	0.488	—	—	1.00 (1.00–1.01)	0.716
**Imaging**										
Atrophy	1.09 (0.64–1.84)	0.750	—	—	1.79 (0.70–4.58)	0.223	—	—	1.91 (0.84–4.34)	0.122
WML	1.41 (0.68–2.94)	0.353	—	—	1.46 (0.61–3.50)	0.398	—	—	0.89 (0.39–2.04)	0.775
High CS-EPVS (>20)	6.15 (2.85–13.20)	<0.001	5.77 (2.21–15.00)	<0.001	4.01 (1.70–9.49)	0.001	2.64 (1.03–6.74)	0.042	1.39 (0.62–3.15)	0.425
High BG-EPVS (>20)	1.31 (0.52–3.30)	0.560	—	—	1.60 (0.50–5.13)	0.426	—	—	0.78 (0.27–2.31)	0.658
**Medical history**										
Pulse steroid therapy	0.63 (0.27–1.46)	0.284	—	—	0.92 (0.35–2.44)	0.872	—	—	0.97 (0.37–2.56)	0.958
Average PSL dose	1.01 (0.97–1.05)	0.778	—	—	1.00 (0.95–1.04)	0.807	—	—	1.00 (0.95–1.04)	0.834
**Medical comorbidities**										
Hypertension	2.56 (1.07–6.08)	0.034	4.47 (1.51–13.20)	0.006	2.66 (0.85–8.30)	0.092	3.26 (0.87–12.20)	0.080	0.34 (0.11–1.06)	0.062
Hyperlipidemia	1.69 (0.48–5.91)	0.413	—	—	0.93 (0.24–3.69)	0.922	—	—	0.98 (0.25–3.85)	0.974
Diabetes mellitus	1.67 (0.40–6.97)	0.484	—	—	2.64 (0.32–22.00)	0.370	—	—	0.83 (0.16–4.21)	0.821
**Blood examinations**										
WBC	0.93 (0.82–1.07)	0.328	—	—	0.95 (0.82–1.10)	0.494	—	—	0.96 (0.82–1.12)	0.594
PLT	1.00 (0.99–1.00)	0.241	—	—	1.00 (0.99–1.00)	0.191	—	—	1.00 (0.99–1.00)	0.831
ESR	1.02 (1.01–1.04)	<0.001	1.01 (1.00–1.03)	0.047	1.02 (1.01–1.04)	0.004	1.02 (1.00–1.03)	0.086	0.99 (0.98–1.00)	0.186
CH50	0.95 (0.92–0.97)	<0.001	0.95 (0.92–0.98)	<0.001	0.99 (0.96–1.01)	0.269	—	—	1.01 (0.99–1.03)	0.487
Anti-dsDNA antibody	1.01 (1.00–1.02)	0.006	1.01 (1.00–1.01)	0.068	1.01 (1.00–1.01)	0.014	1.00 (1.00–1.01)	0.127	1.00 (0.99–1.00)	0.853

Abbreviations: SLEDAI = Systemic Lupus Erythematosus Disease Activity Index; BILAG = British Isles Lupus Assessment Group; nBILAG = neuro British Isles Lupus Assessment Group; WML = white matter lesion; CS = centrum semiovale; EPVS = enlarged perivascular spaces; BG = basal ganglia; PSL = prednisolone; WBC = white blood cell; PLT = platelet; ESR = erythrocyte sedimentation rate; CH50 = 50% hemolytic unit of complement; anti-dsDNA antibodie = anti-double stranded DNA antibody.

Table 4 shows the results of the univariate and multivariate logistic analyses to determine the factors associated with high CS-EPVS. The univariate analysis revealed that increasing age and the severities, as indicated by at the SLEDAI and BILAG scores, were significantly associated with high CS-EPVS (p < 0.001 for SLEDAI and p = 0.002 for BILAG scores). High CS-EPVS was not associated with the nBILAG score and the duration of SLE. The multivariate logistic regression analyses showed that increasing age and disease severity, as indicated by the SLEDAI and BILAG scores, were independently associated with high CS-EPVS (p < 0.001 for SLEDAI and p = 0.009 for BILAG).

**Table 4:** The univariate and multivariate logistic analyses for the presence of high CS-EPVS.

	univariate analysis		SLEDAI		BILAG	
			multivariate analysis		multivariate analysis	
	OR (95% CI)	p value	OR (95% CI)	p value	OR (95% CI)	p value
**Characteristics**						
Age	1.06 (1.03–1.09)	<0.001	1.07 (1.03–1.11)	<0.001	1.07 (1.03–1.12)	<0.001
Smoking	0.82 (0.28–2.41)	0.717	—	—	—	—
Obesity	0.39 (0.11–1.39)	0.146	—	—	—	—
Duration	1.00 (0.99–1.00)	0.815	—	—	—	—
**Imaging**						
Atrophy	1.91 (1.04–3.54)	0.038	2.70 (1.28–5.71)	0.009	2.22 (1.06–4.63)	0.034
WML	2.18 (1.02–4.66)	0.046	1.32 (0.49–3.53)	0.579	1.32 (0.51–3.40)	0.569
**Medical history**						
Pulse steroid therapy	1.17 (0.50–2.75)	0.715	—	—	—	—
Average PSL dose	0.99 (0.96–1.03)	0.776	—	—	—	—
**Medical comorbidities**						
Hypertension	1.10 (0.49–2.47)	0.824	—	—	—	—
Hyperlipidemia	2.36 (0.61–9.17)	0.214	—	—	—	—
Diabetes mellitus	2.73 (0.54–13.70)	0.222	—	—	—	—
**Acivity index**						
SLEDAI score	6.15 (2.85–13.20)	<0.001	5.81 (2.20–15.30)	<0.001	—	—
BILAG score	4.01 (1.70–9.49)	0.002	—	—	4.12 (1.43–11.90)	0.009
nBILAG score	1.39 (0.62–3.15)	0.425	—	—	—	—
**Blood examinations**						
WBC	0.91 (0.79–1.04)	0.157	0.91 (0.74–1.11)	0.352	0.91 (0.75–1.10)	0.494
PLT	1.00 (0.99–1.00)	0.051	1.00 (0.99–1.00)	0.635	1.00 (0.99–1.00)	0.714
ESR	1.01 (1.00–1.02)	0.049	1.00 (0.98–1.01)	0.562	1.00 (0.98–1.01)	0.697
CH50	0.98 (0.96–1.00)	0.135	—	—	—	—
Anti-dsDNA antibody	1.01 (1.00–1.01)	<0.001	1.01 (1.00–1.01)	0.063	1.01 (1.00–1.01)	0.009

Abbreviations: CS = centrum semiovale; EPVS = enlarged perivascular spaces; SLEDAI = Systemic Lupus Erythematosus Disease Activity Index; BILAG = British Isles Lupus Assessment Group; WML = white matter lesion; PSL = prednisolone; nBILAG = neuro British Isles Lupus Assessment Group; WBC = white blood cell; PLT = platelet; ESR = erythrocyte sedimentation rate; CH50 = 50% hemolytic unit of complement; anti-dsDNA antibodie = anti-double stranded DNA antibody.

## Discussion

The novel finding in this study is that the number of semiovale EPVS, as measured by MRI in a large sample of 130 SLE patients, was independently and significantly associated with disease activity (SLEDAI and BILAG scores) – which is consistent with the hypothesis that EPVS are related to neuroinflammatory activity. In addition, our logistic analyses revealed that EPVS in SLE patients could not be explained by either aging or brain atrophy alone, which have been identified as conditions that are associated with the presence of EPVS^5,24^. The exact causes of EPVS are uncertain; however, the PVS is an important conduit for the drainage of interstitial fluid to the ventricles^25^ and can be affected by various factors, including abnormalities at blood brain barrier (BBB)^26,27^. In SLE patients, antibodies bind to the endothelial cells in the vascular wall, influencing the BBB and allowing inflammatory agents to enter the CNS due to increased BBB permeability^28,29^. Evidence of inflammation is present, including vasculitis and the presence of perivascular microglia surrounding small blood vessels^30^. We therefore hypothesize that EPVS are associated with increased BBB permeability or BBB dysfunction due to inflammation in patients with SLE.

Another important finding in our study was that high CS-EPVS were not associated with the presence of neuropsychiatric symptoms (as indicated by the nBILAG score). The current results suggest that CS-EPVS reflect the presence of reactive changes in the WM due to neuroinflammatory activity, but not due to any WM dysfunction. Another point to consider is that CS-EPVS may be present prior to the manifestation of neuropsychiatric symptoms. Our hypothesis may be supported by previous studies which show that indicators of inflammation, such as serum autoantibodies, are present in SLE patients without neuropsychiatric symptoms^31^. In fact, the serum markers of inflammation have been shown to develop 10 years before the diagnosis of SLE, and are likely to be present 1.5 years before the manifestation of any symptoms of the disease^31^. Thus, with regard to the neuropsychiatric manifestations, SLE may be a silent disease, which has the potential to show potential neuroinflammatory activity.

A previous longitudinal study to investigate the short-term fluctuations in PVS volume demonstrated that EPVS were associated with the breakdown of the BBB in multiple sclerosis patients and that they resolved as active inflammation subsided^24^. In this study, high CS-EPVS were not associated with the duration of SLE, suggesting that the EPVS may be a reversible change. However, this suggestion should be also confirmed in a prospective longitudinal study.

Brain changes in SLE patients have been detected using several radiographic techniques, including positron emission tomography (PET)^32^, single-photon emission computed tomography (SPECT)^33,34^, and advanced MRI techniques, such as quantitative susceptibility mapping (QSM)^35^, and MR spectroscopy^36^. Although these neuroimaging studies suggest the usefulness of radiographic techniques for the diagnosis of SLE or the monitoring neuropsychiatric manifestations in SLE patients, the authors have not evaluated relationship between imaging parameters and disease activity in SLE. To our knowledge, there has only been one study to evaluate an imaging biomarker for the disease activity in SLE^3^. In this study using PET, a strong association was found between the SLEDAI score and increased 18FDG uptake in the WM of SLE patients. Because inflammation increases the expression of glucose transporter, the authors interpreted the hypermetabolism of the WM as evidence of acute inflammation. However, PET is limited by its rigid technical requirements and high cost^37^. Thus, our assessment with conventional MRI has clinical significance in that it may provide a simple method for the investigating the disease activity in SLE patients.

Several studies have demonstrated the presence of BG abnormalities in SLE patients. Lim *et al*. demonstrated that the N-acetylaspartate to creatine ratio (NAA/Cr) was decreased in the BG of SLE patients^38^. In a SPECT study, the most common finding among SLE patients was moderate to severe perfusion defects in the BG^39^. However, in the present study, the presence of BG-EPVS was not associated with disease activity (as shown by the SLEDAI and BILAG scores). One possible explanation is that, in comparison to the PVS changes in the BG, those in the CS were more sensitive to inflammation; however, the reason for this is unclear. Our data are also supported by a previous study, which showed no significant difference in the number of PVSs in the regions of the BG between patients with multiple sclerosis and controls, while the number of PVSs in high convexity areas was significantly greater in the patients with multiple sclerosis than in the controls^40^.

Although the previous studies have often shown associations of PVSs with cardiovascular risk factors such as hypertension and diabetes mellitus^41–43^, we found no significant relationship between them. Our results are consistent with the recent study using 7 T MRI that demonstrated EPVS was related to cerebral small vessel disease markers (WMH and cerebral microbleeds), but not to vascular risk factors (hypertension, total cholesterol, and diabetes mellitus)^44^. Moreover, another study reported that correlations between VRS volumes and obesity (body mass index) was not significant^45^. For the hypertension, one possible explanation may be difference across etiology between SLE hypertension and essential hypertension. The previous investigator reported various mechanisms in SLE hypertension, which included the renin-angiotensin system, endothelin, oxidative stress, sex steroids, and metabolic changes^46^. The present study showed that brain atrophy and multifocal WM lesions were not significantly correlated with the disease activity. A main explanation for this may be that these MR findings represent the late-stage pathology, but not the acute-stage pathology. Although brain atrophy and multifocal WM lesions are frequently observed on the MR images of SLE patients^4,47,48^, these findings are more common in the chronic state of the disease^49^. However, this suggestion should be also confirmed in a prospective longitudinal study, because some of the WM lesions in the present study also included acute lesions.

Our study has some limitations that should be acknowledged when interpreting the results. First, we had a relatively small sample of patients recruited from only one institution, which may represent sampling bias. Moreover, the sample size prevented us from exploring a subgroup analysis for the categories of SLEDAI. For instance, there was only one patients with the factor “vasculitis” in the SLEDAI (ulceration, gangrene, tender finger nodules, periungual, infarction, splinter hemorrhages, or biopsy or angiogram proof of vasculitis), although the vasculitis may be more possible affecting the EPVS than other categories. Second, it was limited by its retrospective nature. Because many patients received therapy with antiplatelet, antihypertensive, and antilipemic drugs before undergoing MR imaging, we cannot rule out the possibility that these treatments affected the MR findings, including brain atrophy and the presence of EPVS. Third, this single-center study included only Asian population (Japanese). This homogeneity of the study population should be considered for interpretation of our results because the previous investigation of SLE revealed that genetic associations differ between Caucasians and Asians, and even within different Asian populations^50^. Fourth, we excluded patients with brain abnormalities on MRI such as the large territory infarction and WMH (Fazekas grade 3) to make investigations simple. Thus, our study would include few of patients with severe neuropsychiatric symptoms (high nBILAG scores), which could affect the results in the relationship between CS-EPVS and nBILAG scores. Finally, we used the EPVS scale, which has been widely applied in previous studies. However, this scale may require further validation and reliability studies.

In conclusion, CS-EPVS in SLE patients are specifically associated with the systemic disease activity, but not the nBILAG score. Our results suggest that the presence of CS-EPVS is indicative of reactive changes in the WM that occur due to the neuroinflammatory activity, but not WM dysfunction. Our findings may also provide a new explanation for the pathophysiological mechanisms in the SLE brain; the neuroinflammatory changes in the WM may be present prior to the neuropsychiatric symptoms. Future research should be performed to investigate the longitudinal associations among CS-EPVS, disease activity, clinical symptoms, and the prognosis of SLE to fully ascertain whether CS-EPVS can be used as an imaging biomarker for the monitoring of disease activity, an early indicator of SLE, and also as a prognostic predictor in SLE patients.
